# Local production of the chemokines CCL5 and CXCL10 attracts CD8^+^ T lymphocytes into esophageal squamous cell carcinoma

**DOI:** 10.18632/oncotarget.4617

**Published:** 2015-07-16

**Authors:** Jinyan Liu, Feng Li, Yu Ping, Liping Wang, Xinfeng Chen, Dan Wang, Ling Cao, Song Zhao, Bing Li, Pawel Kalinski, Stephen H. Thorne, Bin Zhang, Yi Zhang

**Affiliations:** ^1^ Biotherapy Center, The First Affiliated Hospital of Zhengzhou University, Zhengzhou, Henan, P.R. China; ^2^ Department of Oncology, The First Affiliated Hospital of Zhengzhou University, Zhengzhou, Henan, P.R. China; ^3^ Department of Thoracic surgery, The First Affiliated Hospital of Zhengzhou University, Zhengzhou, Henan, P.R. China; ^4^ Engineering Key Laboratory for Cell Therapy of Henan Province, Zhengzhou, Henan, P.R. China; ^5^ Department of Surgery, University of Pittsburgh Cancer Institute, University of Pittsburgh, Pittsburgh, PA, USA; ^6^ Robert H. Lurie Comprehensive Cancer Center, Department of Medicine-Division of Hematology/Oncology, Northwestern University Feinberg School of Medicine, Chicago, IL, USA

**Keywords:** esophageal squamous cell carcinoma, T lymphocyte, chemotaxis, CCL5, CXCL10

## Abstract

Esophageal squamous cell carcinoma (ESCC) is a very common malignant tumor with poor prognosis in China. Chemokines secreted by tumors are pivotal for the accumulation of CD8^+^ T lymphocytes within malignant lesions in several types of cancers, but the exact mechanism underlying CD8^+^ T lymphocyte homing is still unknown in ESCC. In this study, we revealed that, compared with marginal tissues, the expression of both chemokine (C-C motif) ligand 5 (CCL5) and (C-X-C motif) ligand 10 (CXCL10) was upregulated in ESCC tissues. CCL5 expression was positively associated with the overall survival of patients. Meanwhile, RT-PCR data showed that the expression of CCL5 and CXCL10 was positively correlated with the local expressions of the CD8^+^ T lymphocyte markers (*CD8* and *Granzyme B*) in tumor tissues. Correspondingly, CD8^+^ T lymphocytes were more frequently CCR5- and CXCR3-positive in tumor than in peripheral blood. Transwell analysis showed both CCL5 and CXCL10 were important for the chemotactic movement of CD8^+^ T lymphocytes. Our data indicate that CCL5 and CXCL10 serve as the key chemokines to recruit CD8^+^ T lymphocytes into ESCC tissue and may play a role in patient survival.

## INTRODUCTION

Chemokines are small heparin-binding proteins weighing 8–14 kDa, which guide the migration of circulating lymphocytes to specific tissues [1]. Relying on specific receptors, chemokines induce the movement of lymphocytes in a concentration-dependent manner [2]. In cancer patients, the immune cell-infiltration is tightly associated with chemotaxis, and the numbers and types of lymphocytes recruited are determined by the local chemokines secreted [3]. As reported, CCL5 and CXCL10 attract CD8^+^ T lymphocytes into various tumors [4–7]. In addition, Muthuswamy et al. [8] and Berghuis et al. [9] reported that CCL5 and CXCL10 favor the recruitment of CD8^+^ T lymphocytes into malignant tissues. However, the expression of CCL5 and CXCL10 and its impact on the migration of CD8^+^ T lymphocytes remain unknown in patients with esophageal squamous cell carcinoma (ESCC).

As the major histologic subtype of esophageal cancer, ESCC, is one of the most common malignancies with high incidence in China [10, 11]. Although substantial improvements have been made in the diagnosis and treatment of ESCC, the 5-year survival after surgery is about 25%. Considering about 66% of patients are inoperable at diagnosis, the 5-year survival of all ESCC patients is just 10% [12]. Hence, there is a need for more promising therapeutic regimens. T cell or Cytokine-induced killer cell (CIK) therapy could be a great help for ESCC treatment. A prerequisite for effective immune cell therapy is efficient delivery of tumor-killing cells into the tumor lesions. Depending on the specific interaction of chemokines with cognitive receptors, T lymphocytes move across the vascular endothelium and enter tumor tissues, where they are retained [13]. Enhancing the specific interaction would improve T lymphocyte infiltration of tumors [14]. Similarly, the tumor-targeted movement of CIK cells depends on the chemokine-chemokine receptor interaction [15]. Therefore, investigating the chemokines and cognitive receptors harboring T lymphocytes within tumors could be beneficial for the improvement of immune cell-based ESCC therapy.

In this project, we focused on the chemotactic movement of CD8^+^ T lymphocytes into ESCC, explored the expression of CCL5 and CXCL10, and investigated the corresponding chemokine receptors (CCR5 and CXCR3) on CD8^+^ T lymphocytes. Our findings show that CCL5-CCR5 and CXCL10-CXCR3 axes are critical for tumor-directed movement of CD8^+^ T lymphocytes in ESCC.

## RESULTS

### Expression of CCL5 and CXCL10 is upregulated in tumor tissues

**Figure 1:**
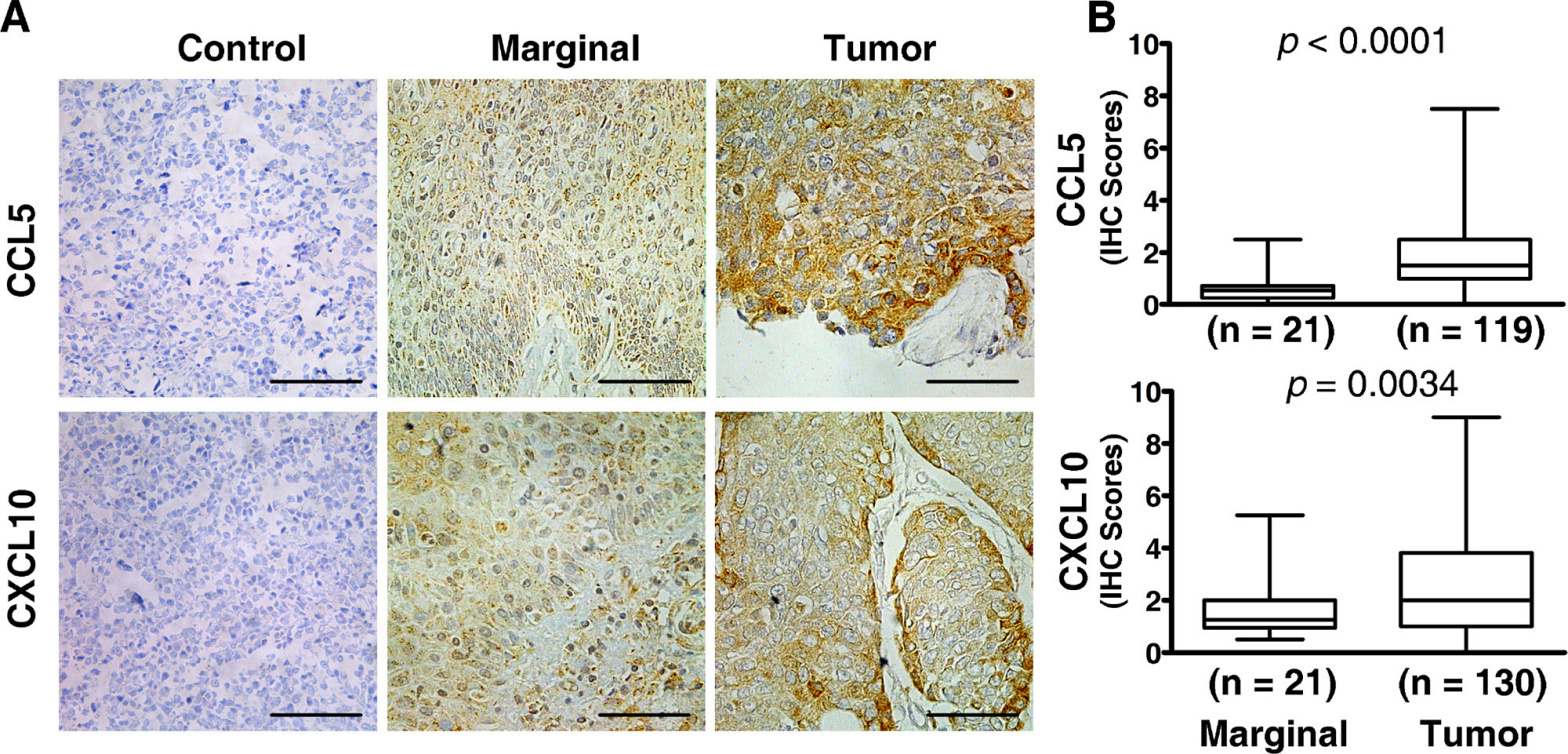
Elevated expression of CCL5 and CXCL10 in ESCC tissue Both marginal and tumor tissues from ESCC patients were examined by immunohistochemistry for the expression of chemokines of interest, and the immunohistochemical scores were calculated as described in the Materials and Methods. **A.** Representative photographs of marginal and tumor tissue sections stained with specific primary antibodies or non-immune rabbit IgG are shown. **B.** Expression scores of CCL5 and CXCL10 were significantly higher in malignant tissues than in marginal tissues. Tumor tissue sections stained with non-immune rabbit IgG were used as a negative control. Scale bar, 50 μM.

To investigate the expression of CCL5 and CXCL10, we performed immunohistochemistry assay (IHC) on tissue sections from ESCC patients. As shown in Figure 1A, CCL5 and CXCL10 were strongly expressed in the cytoplasm of malignant cells, while moderately stained in marginal tissues. Statistical analysis of those samples indicated that both CCL5 and CXCL10 expression (IHC scores) was significantly higher in tumor lesions versus marginal tissues (1.878 ± 1.679 vs. 0.588 ± 0.543, *p* < 0.0001; 2.472 ± 1.997 vs. 1.588 ± 1.042, *p* < 0.005; Figure 1B).

### Association of CCL5 and CXCL10 with clinical factors and overall survival

**Figure 2:**
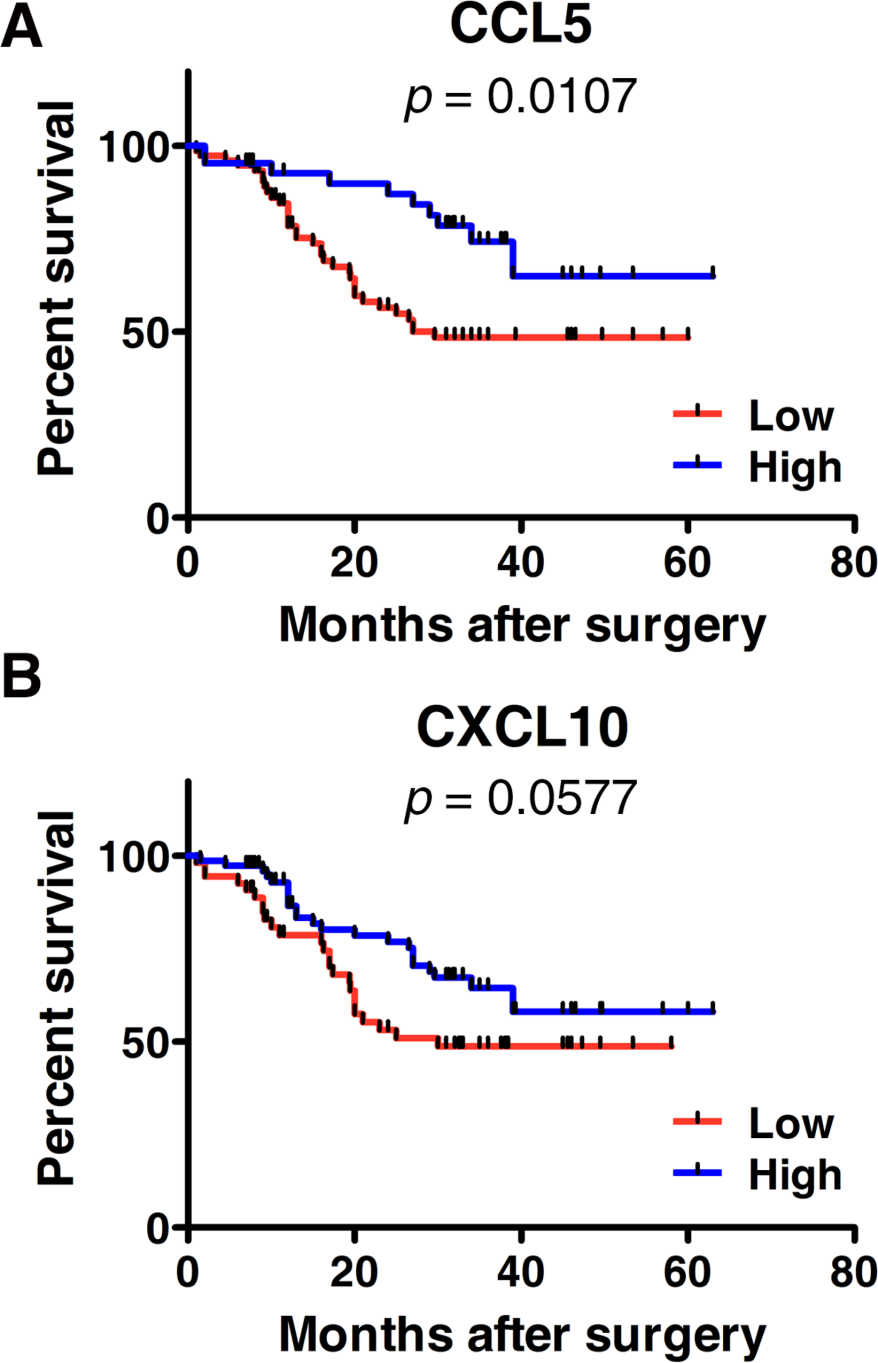
Kaplan-Meier survival curves according to the expressions of CCL5 A. or CXCL10 B A: A log-rank test was performed to compare the overall survival of patients with high (*n* = 76) or low CCL5-expression (*n* = 43). B: A log-rank test was performed to compare the overall survival of patients with high (*n* = 54) or low CXCL10-expression (*n* = 76).

Next, we checked the associations of CCL5 and CXCL10 with various clinical and pathologic characteristics in individual ESCC patients. Those parameters included gender (male, female), age (44–84 y), tumor- node-metastasis stage (TNM, stage I–IV), tumor invasion (T1–T4), differentiation (well, poor), lymph node metastasis (negative, positive), and tumor length (0.5–9 cm). We found the expression levels of CCL5 were significantly elevated in T3–T4 stage patients, compared with their T1–T2 stage counterparts (2.196 ± 2.009 vs. 1.555 ± 1.193, *p* < 0.05) (Table 1). However, the expression of CCL5 was not related to the other clinical factors including gender, age, clinical TNM stage, tumor differentiation, lymph node metastasis, or tumor length (Table 1). Unexpectedly, CXCL10 was also not associated with those parameters (Table 1). In addition, we analyzed the effect of CCL5 and CXCL10 on patients' survival. Patients with high CCL5-expression had greater survival than those with low CCL5-expression (*p* < 0.05) (Figure 2A). As shown in Figure 2B, subjects with high CXCL10-expression exhibited increased survival, although the difference was not statistically significant (*p* > 0.05).

**Table 1:** Clinical and pathological profile of ESCC patients

	*N*	%
Total	207	100
Gender		
Male	144	69.57
Female	63	30.43
Age		
> 60	126	60.87
≤ 60	81	39.13
Clinical stage		
I–IIa	143	69.08
IIb–IV	64	30.92
Differentiation		
Poor (3–4)	88	42.51
Well (1–2)	119	57.49
Lymph node metastasis		
Negative	150	72.46
Positive	57	27.54

### Correlation of CCL5 and CXCL10 expression with CD8^+^ T lymphocyte markers

**Figure 3:**
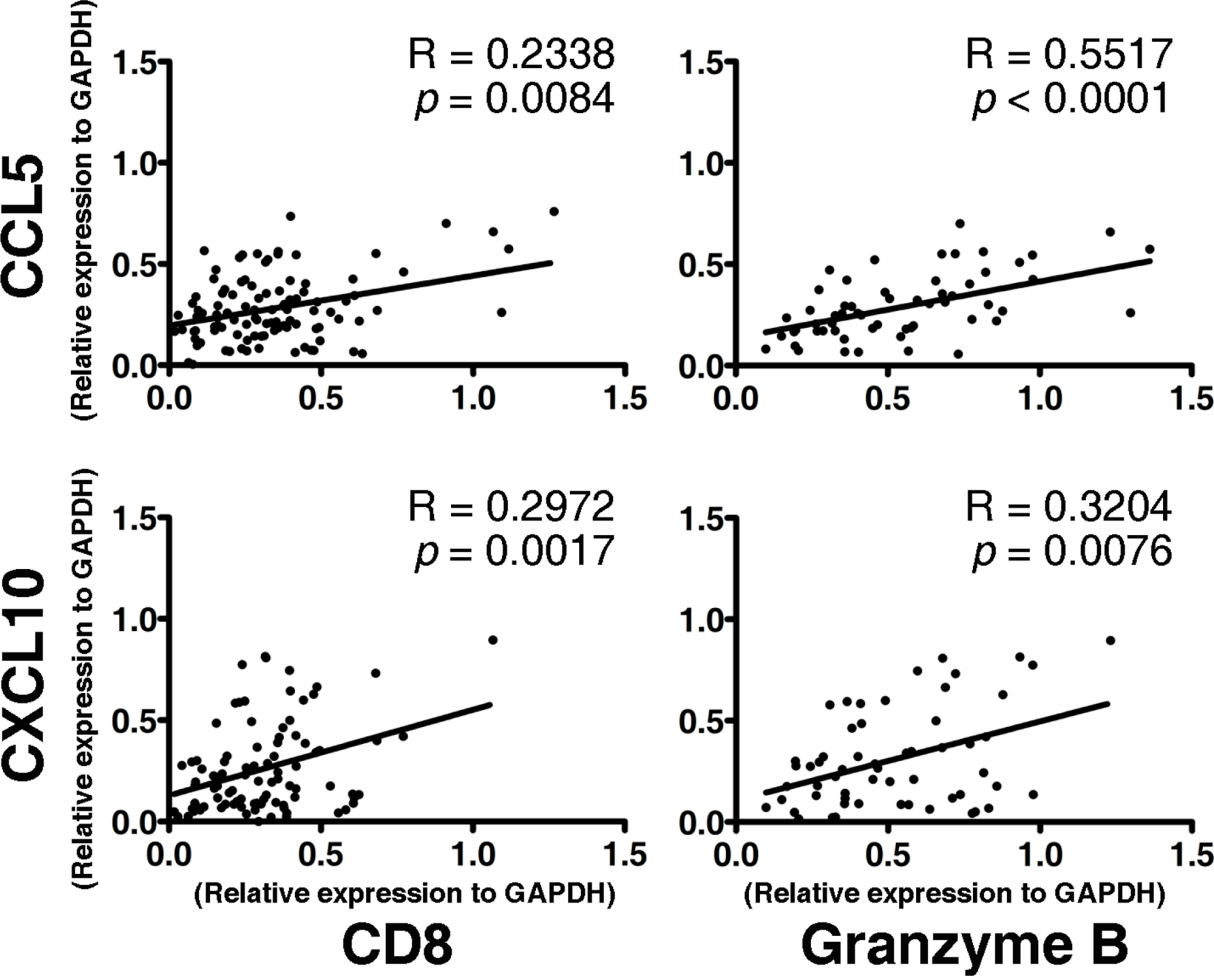
Association between the expression of CD8^+^ effector T lymphocyte markers (CD8 and Granzyme B) and the intensities of CCL5 (upper panel) and CXCL10 (lower panel) in tumor lesions A total of 104 fresh tumor tissues from ESCC patients were processed, RNA was extracted, and an RT-PCR assay of interested genes was performed. The Spearman test was performed to determine the correlation between genes.

To check whether CCL5 and CXCL10 exert an impact on the tumoral accumulation of CD8^+^ T lymphocytes, we performed an RT-PCR assay. In tumor lesions, the local expression of CCL5 and CXCL10 was positively associated with the expression of the CD8^+^ T lymphocyte markers CD8 and Granzyme B (R_CD8_ = 0.2338, *p*_CD8_ < 0.01, R_GranB_ = 0.5517, *p*_GranB_ < 0.001; R_CD8_ = 0.2972, *p*_CD8_ < 0.005, R_GranB_ = 0.3204, *p*_GranB_ < 0.001) (Figure 3). Meanwhile, neither of those two chemokines was correlated with the markers for CD4^+^ T lymphocytes (CD4) or natural killer (NK) cells (CD56) (R_CD4_ = 0.1195, *p*_CD4_ > 0.05, R_CD56_ = 0.1496, *p*_CD56_ > 0.05; R_CD4_ = 0.1533, *p*_CD4_ > 0.05, R_CD56_ = 0.1768, *p*_CD56_ > 0.05) (Supplementary Figure S1). These data demonstrate that CCL5 and CXCL10 predict the recruitment and retention of CD8^+^ T lymphocytes in ESCC.

### Enrichment of CCR5^+^CD8^+^ and CXCR3^+^CD8^+^ T lymphocytes in Tumor-infiltrating Lymphocytes (TILs)

**Figure 4:**
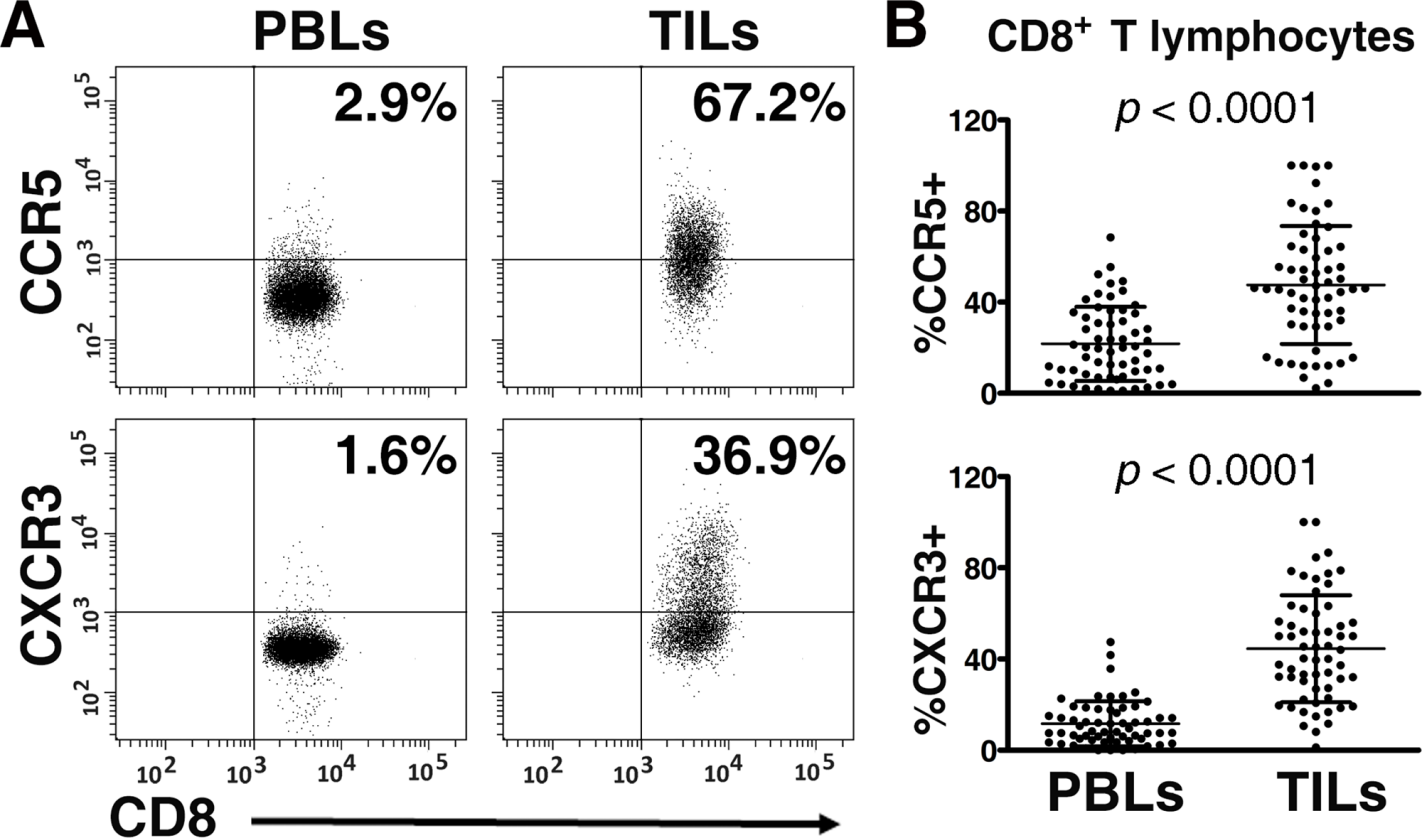
Expression of CCR5 and CXCR3 on CD8^+^ T lymphocytes in peripheral blood lymphocytes (PBLs) and matched tumor-infiltrating lymphocytes (TILs) (*n* = 61) Paired PBLs and TILs were stained with antibodies specific for CD3, CD8, CCR5, and CXCR3. CD8^+^ T lymphocytes (CD3^+^CD8^+^) were gated; then, the percentages of CCR5^+^CD8^+^ and CXCR3^+^CD8^+^ T lymphocytes were determined with multicolor flow cytometry. **A.** Representative plots of CCR5 and CXCR3 staining on CD8^+^ T lymphocytes in matched PBLs and TILs, respectively, are shown. **B.** Paired analysis, for which the paired *t* test was used, showed that CCR5 and CXCR3 were expressed on greater portions of CD8^+^ T lymphocytes in TILs, compared with their counterparts in PBLs.

A specific receptor is a key component in chemokine-driven migration of T lymphocytes [4, 5]. Therefore, we detected the expression of CCR5 (CCL5-specific receptor) and CXCR3 (CXCL10-specific receptor) on CD8^+^ T lymphocytes in TILs and peripheral blood lymphocytes (PBLs). As shown in Figure 4A, CD8^+^ T lymphocytes (CD3^+^CD8^+^) were gated, and the expression of CCR5 and CXCR3 were determined. In 61 paired samples, CCR5 was expressed on a greater fraction of CD8^+^ T lymphocytes in TILs than in PBLs (47.57 ± 25.91% vs. 21.73 ± 16.25%, *p* < 0.0001) (Figure 4B). Similarly, the frequency of CXCR3 was significantly higher on tumor-infiltrating CD8^+^ T lymphocytes than their circulating counterparts (44.52 ± 23.44% vs. 11.62 ± 9.86%, *p* < 0.0001) (Figure 4A and 4B).

### CCL5 and CXCL10 are important for the chemotactic migration of CD8^+^ T lymphocytes

**Figure 5:**
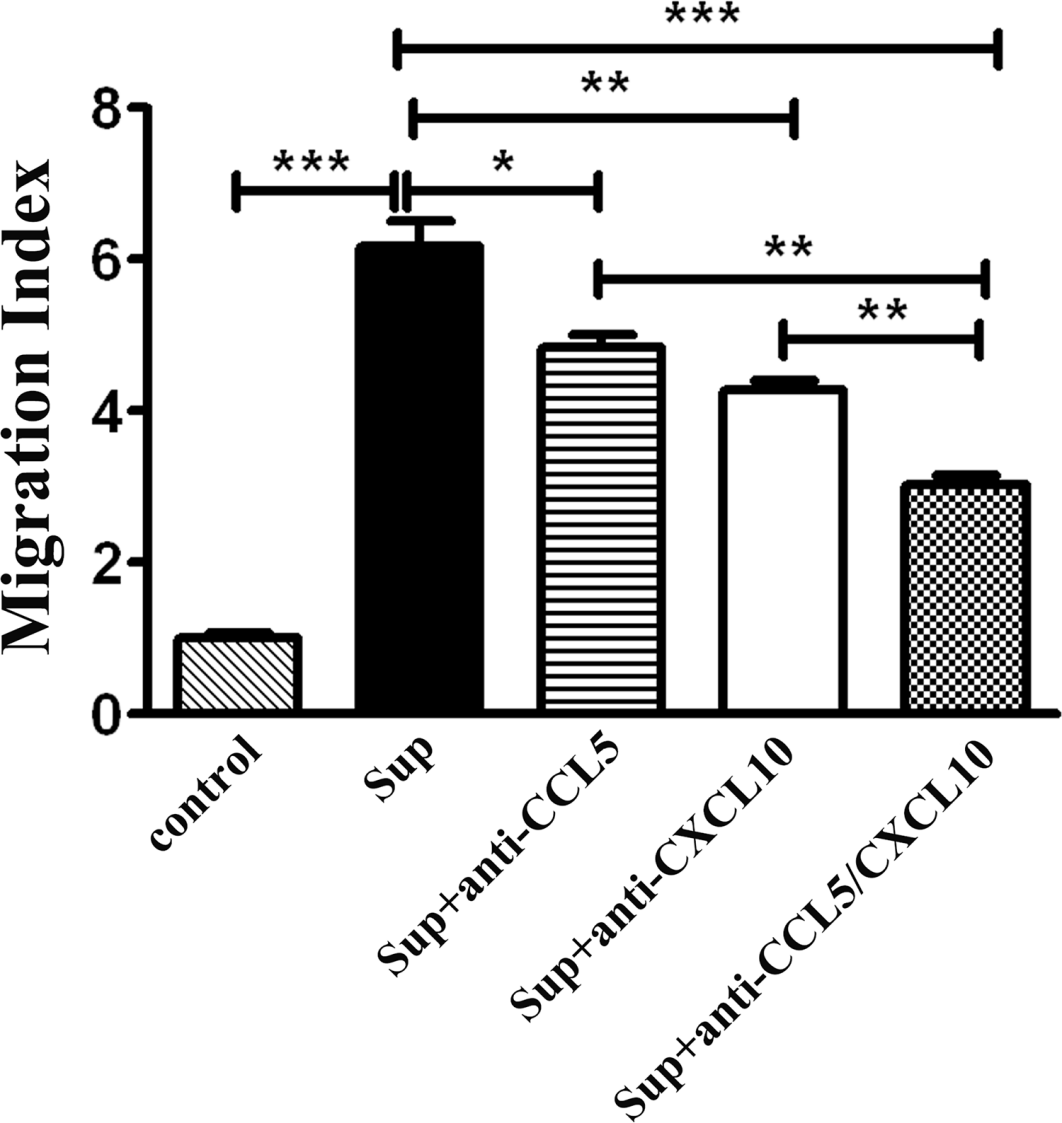
CCL5 and CXCL10 attracting CD8^+^ T lymphocytes *in vitro* Supernatants of primary tumor tissues were added, alone or with neutralizing antibodies specific for CCL5 (1 μg/mL) and/or CXCL10 (5 μg/mL), as indicated. DMEM media with 10% FBS were used as a control. Then, purified CD8^+^ T lymphocytes (Purity > 90%) from the TILs of an ESCC patient were placed into transwell inserts (5-μm pore size). After incubation, cells that migrated into the lower chamber were collected and counted. The migration index was calculated by dividing the number of cells that migrated in indicated groups by the number migrating in control groups. Data shown represent mean ± SD of data from 1 representative experiment of 6 independent experiments. *p < 0.05; **p < 0.01; ***p < 0.001.

To ascertain the regulatory effects of CCL5 and CXCL10 on CD8^+^ T lymphocyte migration, transwell assays were performed. Fresh ESCC tissues were cultured in DMEM media supplied with 10% FBS, and the supernatants were collected 48 h later. In addition, CD8^+^ T lymphocytes were magnetically isolated from fresh TILs. The purity of T lymphocytes used was greater than 90% (Data not shown). Compared with DMEM media supplemented with 10% FBS, the supernatants derived from primary tumor tissues robustly enhanced the movement of T lymphocytes (*p* < 0.001) (Figure 5). However, the neutralizing antibodies for CCL5 or CXCL10 significantly hampered the migration induced by tumor supernatant (*p_CCL5_* < 0.05; *p_CXCL10_* < 0.01) (Figure 5), and the migration of T lymphocytes was further inhibited by the combined use of anti-CCL5 and anti-CXCL10 neutralizing antibodies (*p* < 0.001) (Figure 5). Similar results of inhibited movement of CD8^+^ T lymphocytes by CCL5- and/or CXCL10-specific antibodies were obtained in five other independent experiments (Supplementary Figure S2). These results indicated that both CCL5 and CXCL10 are important in attracting CD8^+^ T lymphocytes towards ESCC.

## DISCUSSION

The natural infiltration of T lymphocytes is common in tumors [16–19]; the chemotaxis-induced accumulation of T lymphocytes has great effects on tumor differentiation, metastasis, disease progress, and patient survival [20–24]. Although several studies have examined the expression of chemokine receptors by esophageal carcinoma cells [25, 26], the expressions of ESCC-associated chemokines in relation to local T lymphocytes accumulation had not been investigated. In this study, we sought to determine CCL5 and CXCL10 expression and their effects on CD8^+^ T lymphocyte trafficking to ESCC. Several studies indicated that the tumoral expression of CCL5 is tightly related with CD8^+^ T lymphocytes infiltrating in colorectal carcinoma [8, 27], melanoma [28], and sarcoma [9]. CXCL10 is involved in the recruitment of CD8^+^ T lymphocytes in breast cancer [29], colorectal carcinoma [8], melanoma [28], and hepatocellular carcinoma [30]. Consistent with those reports, we found the expression of CCL5 and CXCL10 in ESCC tissues was a) strongly elevated, compared to surrounding healthy tissues, and b) positively correlated with the local expression of CD8^+^ T lymphocyte markers, including CD8 and Granzyme B. These observations strongly indicate that CCL5 and CXCL10 are involved in the local recruitment of CD8^+^ T lymphocytes to ESCC lesions and their local retention.

To further verify the effects of CCL5 and CXCL10 on tumoral infiltration of CD8^+^ T lymphocytes, we checked the expression of their specific receptors on T lymphocytes. Upon activating specific receptors, chemokines recruit the cells expressing such receptors into tumors [31–33]. Therefore, studying CCR5 (specifically recognizing CCL5) and CXCR3 (specifically recognizing CXCL10) helps to elucidate the molecular basis of CD8^+^ T lymphocyte homing to specific sites. The CCR5-CCL5 axis has been reported to induce the tumoral accumulation of CD8^+^ T lymphocytes in renal cell carcinoma [4], nasopharyngeal carcinoma [5], and colorectal carcinoma [27]. The CXCR3-CXCL10 interaction induces the infiltration of CD8^+^ T lymphocytes in renal cell carcinoma [4] and colorectal carcinoma [27]. In this project, we performed a flow cytometry assay to detect the frequencies of CCR5 and CXCR3 on CD8^+^ T lymphocytes in TILs and PBLs, separately. The percentages of CCR5^+^CD8^+^ and CXCR3^+^CD8^+^ T lymphocytes were 47.57 ± 25.91% and 44.52 ± 23.44% in TILs, respectively, whereas the proportions of those subgroups in T lymphocytes were just 21.73 ± 16.25% and 11.62 ± 9.86% in PBLs. The differences were statistically significant (*p*_CCR5_ < 0.0001;*p*_CXCR3_ < 0.0001). This phenomenon suggests that CCR5 and CXCR3 are activated by their ligands CCL5 and CXCL10 and then deliver CD8^+^ T lymphocytes into malignant tissues, where CCL5 and CXCL10 are produced and the concentrations of chemokines are higher. To consolidate the roles of CCL5 and CXCL10 in attracting T lymphocytes, transwell assays were performed. As expected, the migration of CD8^+^ T lymphocytes induced by tumor supernatant was partially but significantly inhibited by CCL5- and/or CXCL10-neutralizing antibodies. Taking the data together, it can be inferred that the CCL5-CCR5 and CXCL10-CXCR3 axes are critical for the movement of CD8^+^ T lymphocytes to tumor tissues in ESCC patients.

Tumoral infiltration of cytolytic CD8^+^ T lymphocytes is associated with a favorable prognosis and increased survival [34–36]. In our study, CCL5 and CXCL10 were positively correlated with ESCC patient survival (Figure 2). Patients with high CCL5- or CXCL10-expression exhibited better overall survival. The impact of chemokines on survival is likely associated with the accumulation of CD8^+^ T lymphocytes. Consistent with our observations, CCL5 and CXCL10 are reported to recruit antitumor CD8^+^ T lymphocytes into malignancies and are positively associated with the survival of patients with colorectal cancer or hepatocellular carcinoma [30, 37].

Understanding the molecular basis of T lymphocytes accumulation in tumors is important for the improvement of immune cell-based therapy. As the tissue-specific migration of both T and CIK cells is dependent on chemotaxis, the movement of these tumor-killing cells to tumor sites would be enhanced when the interactions between chemokine ligand and receptor are employed by tumor-infiltrating T cells [14, 15, 38]. Recent technical advances in gene modification make it easy to introduce specific receptors, including chemokine receptors into T and CIK cells [14, 39, 40]. Therefore, the key for efficient delivery of tumor-killing cells is to determine which chemokines are involved in immune cells *in vivo*, especially for T lymphocyte trafficking. Considering that immune cells are prone to migrate to sites with higher concentrations of chemokines [2, 3], the chemokines highly expressed in tumor sites are good candidates. The expression of chemokines in tumors is complex. Some chemokines are upregulated in tumor tissues [41–43], while others are downregulated [44, 45]. In our study, CCL5 and CXCL10 were more highly expressed in cancerous tissues than in marginal sites; the chemokines' expression was not affected by patients' age, gender, tumor differentiation, or clinical TNM stages, except that CCL5 was further upregulated in clinical T3-T4 stages (Table 1). These data imply that CCL5 and CXCL10 are ideal candidates for the effective delivery of T and CIK cells.

CCL5 and CXCL10, together with CCR5 and CXCR3, recruit CD8^+^ T lymphocytes into ESCC tissue. Moreover, CCL5 and CXCL10 are overexpressed in malignant lesions, suggesting that CCR5 and CXCR3 induction would enhance the tumoral delivery of infused T and CIK cells and improve the curative efficacy of such adoptive therapy.

## MATERIALS AND METHODS

### Clinical samples

A total of 207 patients with esophageal squamous cell carcinoma were enrolled in this study, which was approved by the Ethics Committee Board of the First Affiliated Hospital of Zhengzhou University. All patients signed informed consent forms. Tumor stage and differentiation was graded according to the classification system of the American Joint Committee on Cancer (AJCC, 6^th^ Edition). The detailed information of patients is listed in Table 2. During surgery from June 2008 through March 2014 in the First Affiliated Hospital of Zhengzhou University, 207 tumors and 61 paired peripheral blood samples were collected. In addition, 21 marginal tissues were sampled.

**Table 2:** Association of CCL5 and CXCL10 scores with clinicopathological factors

	CCL5			CXCL10		
	*N*	Mean ± SD	*p*-value	*N*	Mean ± SD	*p*-value
Gender						
Male	86	1.904 ± 1.748	0.7871	94	2.384 ± 1.905	0.4189
Female	33	1.811 ± 1.508		36	2.701 ± 2.234	
Age						
≤ 60	47	1.888 ± 2.018	0.9578	53	2.486 ± 2.046	0.9463
> 60	72	1.872 ± 1.432		77	2.462 ± 1.977	
Clinical stage						
I–IIa	82	1.902 ± 1.712	0.8155	92	2.395 ± 2.081	0.4963
IIb–IV	37	1.824 ± 1.625		38	2.658 ± 1.793	
T stage						
T1–T2	59	1.555 ± 1.193	**0.0369**	65	2.254 ± 1.617	0.3487
T3–T4	60	2.196 ± 2.009		65	2.566 ± 2.133	
Differentiation						
Poor	59	1.747 ± 1.203	0.4022	62	2.424 ± 1.802	0.7975
Well	60	2.007 ± 2.045		68	2.515 ± 2.173	
Lymph node metastasis						
Negative	87	1.977 ± 1.716	0.2916	97	2.364 ± 2.049	0.2940
Positive	32	1.609 ± 1.570		33	2.788 ± 1.832	
Tumor length						
≤ 3 cm	57	2.013 ± 1.771	0.4027	62	2.477 ± 1.971	0.9781
> 3 cm	62	1.754 ± 1.595		68	2.467 ± 2.036	

### Isolation of TILs and PBLs

Tumor tissues were rinsed with Roswell Park Memorial Institute 1640 (RPMI-1640) media (Invitrogen Life Technologies, Carlsbad, CA, USA) to remove traces of blood and then cut into small pieces. The tissues were incubated with 300 μg/mL collagenase (Roche, Indianapolis, IN, USA) and 50 μg/mL deoxyribonuclease I (Sigma-Aldrich, St. Louis, MO, USA) for 2 h at 37°C. Following that, the samples were mechanically disaggregated. TILs were separated by centrifugation at 2,000 rpm for 20 min on Ficoll-Paque Plus (Sigma-Aldrich, St. Louis, MO, USA). PBLs were isolated from heparinized blood samples by Ficoll-Paque Plus density centrifugation.

### Flow cytometry

TILs and PBLs were suspended in flow buffer (Phosphate-buffered saline containing 2% fetal bovine serum) and incubated with anti-CD3-APC (HIT3a), anti-CD8-APC/Cy7 (HIT8a), anti-CCR5-PE/Cy7 (J418F1), anti-CXCR3-PE (G025H7), or isotype-matched antibodies (Biolegend, San Diego, CA, USA) for 30 min at 4°C. After incubation, cells were rinsed twice. Then, cells were analyzed using a BD CantoII flow cytometer (Becton Dickinson, San Jose, CA, USA).

### RT-PCR

Tumor tissues were cut into 20 mm pieces and mechanically minced. Then, total RNA was extracted using Trizol solution (Invitrogen Life Technologies, Carlsbad, CA, USA). Subsequently, RNA from each sample was separately reverse-transcribed using the PrimeScript RT Reagent Kit (Takara Bio, Otsu, Shiga, Japan). Then, the genes of interest were tested. Primers used were listed in Table 3. The initial step was performed at 95°C for 30 s and the amplification was performed for 30 cycles of 95°C for 15 s, 58°C for 30 s, and 72°C for 30s. PCR products were separated on a 1.5% agarose gel and recorded. Targeted bands were analyzed using ImageJ software (National Institute of Health, USA) and optical densities were calculated. Then, the relative expression of genes was determined. The house-keeping gene GAPDH was used as reference.

**Table 3:** The sequences of primers used

Gene	Sequence
GAPDH	Sense: 5′-GGAGCCAAAAGGGTCATCATCTC-3′
	Anti-sense: 5′-GAGGGGCCATCCACAGTCTTCT-3′
CD8	Sense: 5′-CGCTGTCAGATCCCCTTTGT-3′
	Anti-sense: 5′-GAGGAAGGACCCTCTCCCTT-3′
Granzyme B	Sense: 5′-GCAGGAAGATCGAAAGTGCG-3′
	Anti-sense: 5′-TACAGCGGGGGCTTAGTTTG-3′
CCL5	Sense: 5′-CAGTCGTCTTTGTCACCCGA-3′
	Anti-sense: 5′-TGTAACTGCTGCTGTGTGGT-3′
CXCL10	Sense: 5′-AACTGTACGCTGTACCTGCAT-3′
	Anti-sense: 5′-GCATCGATTTTGCTCCCCTC-3′

### Immunohistochemistry

Formalin-fixed paraffin-embedded sections (3 μm) were de-paraffinized in xylene, rehydrated through graded alcohol, and washed briefly in tap water. Endogenous peroxidase was blocked with methanol containing 0.3% hydrogen peroxide for 30 min. To retrieve antigenicity, sections were boiled in 10 mM citrate buffer (pH 5.8) for 30 min in a microwave oven (800 W). Next, sections were incubated with goat serum diluted in PBS (pH 7.4) for 30 min at room temperature. Subsequently, sections were incubated at 4°C overnight with the primary antibodies specific for CCL5 (dilution 1: 200) or CXCL10 (dilution 1:200) (Abcam, Cambridge, UK). Then, sections were rinsed in fresh PBS and incubated with horseradish peroxidase-linked secondary antibodies at room temperature for 60 min. Finally, sections were stained with 3, 3′-diaminobenzidine (DAB) substrate (Dako, Carpinteria, CA, USA) and counterstained with Mayer's hematoxylin. Non-immune rabbit IgG at the same dilution was used as negative control. Photos were recorded under a microscope (Leica, Wetzlar, Germany).

### Evaluation of immunohistochemical staining

All sections were assessed at 20 × magnification by 2 experienced observers. The staining was evaluated based on the intensity (weak = 1, moderate = 2, and high = 3) of chemokine immunostaining and the density (0% = 0, 1–40% = 1, 41–75% = 2, > 76% = 3) of positive tumor cells. The final score of each sample was multiplied by the intensity and density. If the two evaluations did not agree, the specimens were re-evaluated and then classified according to the assessment given most frequently by the observers. For CCL5 and CXCL10, samples with a score > 1.50 were regarded as having high expression.

### Purification of CD8^+^ T lymphocytes

TILs were isolated from fresh ESCC tissues as mentioned above. Then, CD8^+^ T lymphocytes were further purified by positive selection using CD8 microbeads (Miltenyi Biotec, Auburn, CA, USA) according to the manufacturer's protocol. Briefly, 10^7^ TILs were suspended in magnetic-activated cell sorting (MACS) buffer, incubated with 20 μL CD8 microbeads at 4°C for 15 min, and washed once. Then, CD8^+^ T lymphocytes were magnetically isolated.

### Transwell assay

The chemotactic migration of CD8^+^ T lymphocytes was evaluated in 24-well plates with 5-μm pore size polycarbonate filters (Corning Inc, Coring, NY, USA) [8]. First, ESCC tumor tissues were cut into small pieces and cultured in Dulbecco's Modified Eagle Medium (DMEM) with 10% fetal bovine serum (FBS) for 48 h. Then, 600 μL of tumor supernatants were placed into the lower chambers of transwell plates. Anti-CCL5 (1 μg/mL) and/or anti-CXCL10 (5 μg/mL) neutralizing antibodies were added as indicated. DMEM media with 10% FBS were used as a control. Purified CD8^+^ T lymphocytes from TILs (Purity > 90%) were counted. Then 5 × 10^5^ CD8^+^ T lymphocytes were added into the upper chambers and incubated at 37°C in a 5% CO_2_ atmosphere for 2 h. Cells in the bottom chambers were counted using a limited 60-second analysis on a flow cytometer.

### Statistical analysis

Analyses were performed using GraphPad Prism 5 software (GraphPad Software, La Jolla, CA, USA). Data were expressed as mean ± SD. The Student's *t* test and one-way ANOVA were conducted to compare the differences between variables. The Spearman test was adapted to determine the correlation between chemokine genes and CD8^+^ T lymphocyte-related markers. Kaplan-Meier curves in combination with log-rank tests were used to compare the survival of patients in different groups. Values with *p* < 0.05 (two-tailed) were considered significant.

## SUPPLEMENTARY FIGURES


