# Supplementary material for: Local production of the chemokines CCL5 and CXCL10 attracts CD8+ T lymphocytes into esophageal squamous cell carcinoma
Source: Oncotarget. 2015 Jul 16;6(28):24978–89. doi: 10.18632/oncotarget.4617 (PMC4694808; doi:10.18632/oncotarget.4617)
Supplement: Supplementary file 1 [file oncotarget-06-24978-s001.pdf]

SUPPLEMENTARY FIGURES

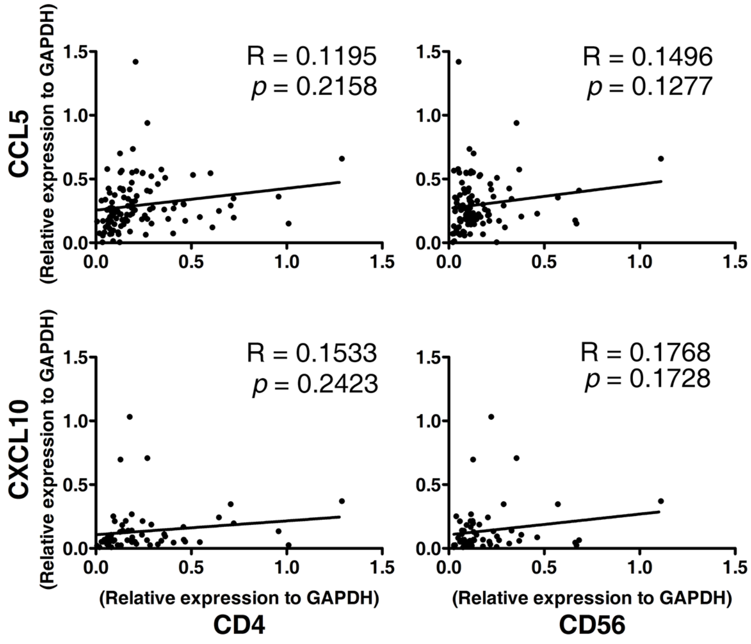

Supplementary Figure S1: Association of the expression of CCL5 (upper panel) and CXCL10 (lower panel) with CD4^{+} T lymphocyte (CD4) or NK cell (CD56) markers in tumor lesions.

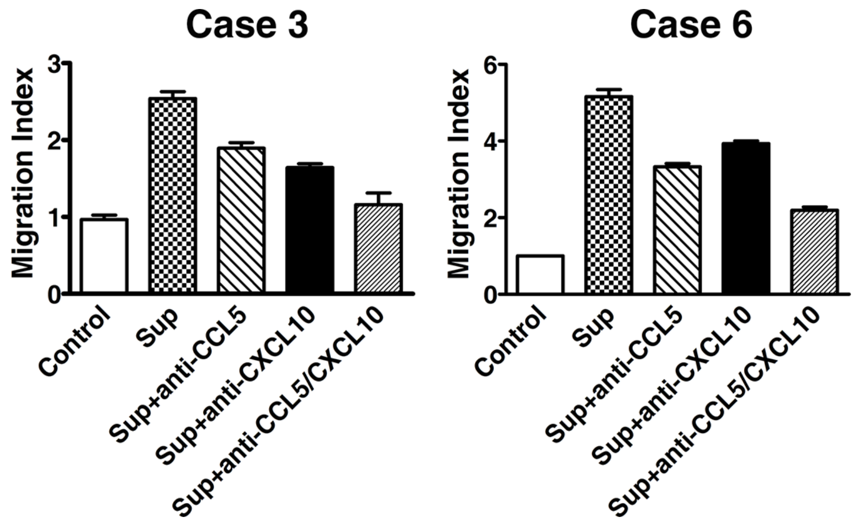

Supplementary Figure S2: CCL5- and/or CXCL10- specific neutralizing antibodies inhibit the migration of CD8^{+} T lymphocytes. Shown are two representative assays from 6 independent experiments.
